# Barriers to post-prostatectomy stress incontinence care: knowledge gaps, patient concerns, and urologist communication

**DOI:** 10.1007/s00345-026-06185-8

**Published:** 2026-01-17

**Authors:** Viktoria Menzel, Christer Groeben, Falk Hoffmann, Felix K.H. Chun, Lothar Weissbach, Christian Thomas, Johannes Huber, Martin Baunacke

**Affiliations:** 1 Department of Urology, TU Dresden, Fetscherstrasse 74, 01307 Dresden, Germany; 2 Department of Urology, University Heidelberg, Im Neuenheimer Feld 110, 69120 Heidelberg, Germany; 3 Department of Health Services Research, Carl Von Ossietzky Universität, Ammerlaender Heerstrasse 140, 26111 Oldenburg, Germany; 4 Department of Urology, Goethe-University Hospital, Theodor-Stern-Kai 7, 60590 Frankfurt, Germany; 5 Health Research for Men gGmbH, Gfm, Claire-Waldoff-Strasse 3, 10117 Berlin, Germany

**Keywords:** Stress urinary incontinence, Continence aid, Male continence care, Patient awareness, Health service research

## Abstract

**Purpose:**

Stress urinary incontinence (SUI) after radical prostatectomy markedly reduces quality of life, yet care gaps remain. This study evaluated patients’ knowledge of treatment options and use of continence aids.

**Methods:**

We analysed follow-up data from the multicentre HAROW study (2013–2018) and a cross-sectional study from Dresden (2021). Included were men up to 15 years after prostatectomy using ≥ 2 pads daily. The survey examined awareness of surgical treatments, continence aids, information sources, and barriers to therapy.

**Results:**

Ninety-nine patients participated (HAROW: 62; Dresden: 37). Median age at surgery was 67 years (47–85); median postoperative interval 11 years (0–15). Continuous leakage was reported by 70% (68/97), and 53% (51/96) used > 3 pads/day. Pads were the main aid (97%, 93/96); condom catheters (12%) and penile clamps (2%) were rarely used, with 86% unaware of these options. Knowledge of surgical treatments was absent in 62% (55/89). Better awareness was linked to younger age (*p* = 0.002) and fewer pads used (*p* = 0.04). Urologists were the main information source (88%), followed by treating hospital (50%) and partners (44%). Key reasons for not seeking surgery were sufficient coping with pads (69%), doubts about efficacy (55%), and fear of health risks (44%).

**Conclusion:**

Most men with SUI after prostatectomy remain poorly informed about surgical options despite frequent urologist consultations. Fear and misconceptions limit therapy uptake. Structured, targeted education is needed to bridge the gap between clinical need and treatment, potentially improving utilization and quality of life.

## Introduction

Stress urinary incontinence (SUI) is one of the most common and burdensome complications following radical prostatectomy (RP), with reported prevalences ranging from 4% to 40% [1–3]. While often underreported, SUI can have a substantial effect on long-term quality of life and poses a major challenge for affected men [4–6]. With the increasing number of RPs, the long-term consequences of treatment, including SUI, are becoming increasingly relevant in both clinical care and health policy [7].

Several studies suggest that a substantial proportion of patients with SUI remain inadequately treated [3, 8]. According to clinical guidelines, surgical interventions should be considered only after at least 12 months of unsuccessful conservative therapy, which typically includes pelvic floor exercises and, in some cases, pharmacological support [5, 9]. Once conservative measures fail, patients with SUI often rely on continence aids such as absorbent pads, condom catheters, or penile clamps to manage symptoms. Furthermore, a range of surgical options are available: slings, adjustable systems or artificial urinary sphincters [10, 11]. Several studies have indicated that men with stress incontinence after RP, particularly those with high distress, are not receiving adequate treatment. This suggests a gap in care, underlined by recent German data showing declining rates of incontinence surgery despite increasing prostatectomy numbers, and by reports of inadequate counselling on absorbent products [7, 12–14]. This is surprising, given that those affected are already closely monitored by a urologist during their prostate cancer follow-up care, who would typically serve as their primary point of contact. A lack of patient knowledge regarding treatment options and continence aids may contribute to the underutilization of care.

The aim of this study was to evaluate the use of continence aids, patient awareness of treatment possibilities, and reasons why patients do not undergo surgical treatment.

## Materials and methods

This pooled analysis combines follow-up data from the multicentre prospective HAROW study (2013–2018) and a cross-sectional study conducted in 2021 at the University Hospital Dresden, Germany.

The HAROW study (Hormone Therapy, Active Surveillance, Radiation, Operation, or Watchful Waiting) was a non-interventional, prospective observational study conducted between 2008 and 2013, following patients with localized prostate cancer across Germany. Among the total cohort, 1260 patients underwent RP at 114 different institutions, representing one-fourth of all German RP providers [3]. Functional outcomes after RP were reassessed in 2017 (*n* = 936) [15]. Urinary incontinence was defined as ≥ 2 pads/day. Sexual function outcomes were validated according to International Consortium for Health Outcomes Measurement (ICHOM) standards [16], and potency was defined as an erection firm enough for sexual intercourse.

We contacted 525 patients with SUI and/or erectile dysfunction (ED) with interest in sex [12]. Identification of SUI and ED was based on previously collected HAROW follow-up data. Patients with ED had originally been recruited to investigate ED care [17]; however, for the present analysis only men fulfilling the ≥ 2 pads/day criterion were included. Patients were contacted by mail in 2023, with up to two reminders for non-responders. The second dataset derived from a cross-sectional survey at the University Hospital Dresden. All men who undergo RP at the institution are routinely surveyed annually regarding quality of life and functional and oncological outcomes. From this cohort, patients treated with RP between 2015 and 2021 were invited in 2021 to participate in an educational workshop on SUI and completed questionnaires beforehand. were recruited in 2021 for an educational workshop on SUI who received with RP between 2015 and 2021. Prior to this workshop, patients received the questionnaires. Eligible participants were men using ≥ 2 pads and without prior continence surgery.

In both cohorts, only male patients with persistent SUI, defined as the use of more than two pads per day, and no history of prior surgical treatment for incontinence were included in this analysis. Pad use was self-reported. Data collection focused on demographic variables, current use of continence aids (type and quantity), sources of information on continence management, knowledge of surgical treatment options, and perceived barriers to uptake of incontinence surgery. While the questions on the care situation were identical in both cohorts, health-related quality of life was assessed using validated instruments. The Dresden cohort additionally received three items on awareness of continence aids. The PHQ-4 was used as a brief screening tool for anxiety and depression [18], and the Global Health and Quality of Life scales of the EORTC QLQ-C30, a widely applied cancer-specific instrument, were administered [19]. For pooled analyses, only identical items were considered. Numerical denominators varied due to item-level missing data.

Descriptive statistics were applied to summarize the data. Group comparisons between patients with and without prior knowledge of surgical treatment options were performed using chi-square or t-tests, as appropriate. A difference in the data with a p value of < 0.05 was considered statistically significant. Analyses were performed using IBM SPSS Statistics (Version 30).

## Results

### Collective

In the HAROW cohort, 525 patients with ED or incontinence were contacted regarding their treatment. Eighty were lost to follow-up (8 deceased, 72 with unknown address), resulting in a response rate of 68% (304/445). Among these, 62 patients with incontinence were eligible and included. In the Dresden cohort, 37 of 40 patients responded (93%). Altogether, 99 patients were analysed (62 from HAROW, 37 from Dresden).

The median age at surgery was 67 years (47–85). The median time since surgery was 11 years (0–15). 70% (68/97) reported persistent urinary leakage, 53% (51/96) used more than three pads per day, and 22% (21/97) indicated a complete lack of control over urinary loss. The EORTC-C30 item quality of life score was 4.7 ± 1.4 (5 (1–7)), and the global health score was 4.7 ± 1.2 (5 (2–7)) (Table 1).

**Table 1:** Descriptive demographic analysis of the cohort (*p* value of < 0.05 is considered statistically significant)

		Collective (*n* = 99)	HAROW (*n* = 62)	Dresden (*n* = 37)	*p*-value
Age at time of radical prostatectomy (years)	Mean ± SD	66.6 ± 7.1	65.9 ± 7.3	67.9 ± 6.7	0.2
	Median (Min – Max)	67 (47–85)	67 (47–84)	69 (53–85)	
Median time between surgery and survey (years)	Mean ± SD	8.8 ± 5.5	12.8 ± 1.5	2.2 ± 2.5	**< 0.001**
	Median (Min – Max)	11 (0–15)	13 (10–15)	2 (0–10)	
Marital status (*n* = 96)	Single	8 (8%)	5 (8%)	3 (8%)	1.0
	Partnership	88 (92%)	55 (92%)	33 (92%)	
Education level (*n* = 90)	Middle school degree or less	57 (63%)	35 (66%)	22 (59%)	0.5
	High school degree	33 (37%)	18 (34%)	15 (41%)	
Monthly household net income (*n* = 91)	< 1.500 Euro	17 (19%)	9 (16%)	8 (22%)	0.7
	1.500–4.000 Euro	62 (68%)	39 (71%)	23 (64%)	
	> 4.000 Euro	12 (13%)	7 (13%)	5 (14%)	
Health insurance (*n* = 96)	Statutory	79 (82%)	50 (85%)	29 (78%)	0.4
	Private	17 (18%)	9 (15%)	8 (22%)	
Pad usage per day (*n* = 96)	None	8 (8%)	7 (12%)	1 (3%)	**0.03**
	One pad	14 (15%)	4 (27%)	10 (27%)	
	Two pads	23 (24%)	16 (27%)	7 (19%)	
	Three pads or more pads	51 (53%)	32 (54%)	19 (51%)	
Frequency of involuntary urine loss (*n* = 97)	Never	0 (0%)	0 (0%)	0 (0%)	0.1
	Once per week	8 (9%)	8	0 (0%)	
	Twice per week	3 (3%)	1	2 (5%)	
	1x per day	6 (6%)	3	3 (8%)	
	Multiple times a day	12 (12%)	9	3 (8%)	
	Constantly	68 (70%)	39	29 (77%)	
Control of urine loss (*n* = 97)	Complete loss of control	21 (22%)	17 (28%)	4 (11%)	**< 0.001**
	Frequent dribbling	35 (36%)	24 (40%)	11 (30%)	
	Occasional dribbling	35 (36%)	13 (22%)	22 (59%)	
	Complete control	6 (6%)	6 (10%)	0 (0%)	
PHQ-4 (*n* = 89)		2.4 ± 2.8 2 (0–11)	2.1 ± 2.9 1 (0–11)	2.9 ± 2.6 2.5 (0–10)	0.1
EORTC C30 global health (*n* = 96)		4.7 ± 1.2 5 (2–7)	5.0 ± 1.2 5 (2–7)	4.3 ± 1.1 4 (2–7)	**0.01**
EORTC C30 quality of life (*n* = 96)		4.7 ± 1.4 5 (1–7)	5.0 ± 1.4 5 (1–7)	4.3 ± 1.2 4 (1–7)	**0.01**

### Use and awareness of continence aids

90% (86/96) relied on absorbent pads as their primary continence aid, whereas alternative options such as condom catheters (12%, 10/85) and penile clamps (2%, 2/84) were rarely used. The Dresden cohort was surveyed about their awareness of continence aids. Only 19% (7/37) were familiar with condom catheters, and 11% (4/37) knew about penile clamps (Table 2).

**Table 2:** Descriptive analysis of the use of continence aids, prior knowledge of surgical treatment options and information seeking

				Collective (*n* = 99)
Knowledge of incontinence aids (Dresden cohort) (*n* = 37)	Pads		known	37 (100%)
			unknown	0 (0%)
	Condom urinals		known	7 (19%)
			unknown	30 (81%)
	Penile clamp		known	4 (11%)
			unknown	33 (89%)
Usage of incontinence aids	Pads (*n* = 96)		No use	3 (3%)
			I used it at least once	93 (97%)
	Condom urinals (*n* = 85)		No use	75 (88%)
			I used it at least once	10 (12%)
	Penile clamp (*n* = 84)		No use	82 (98%)
			I used it at least once	2 (2%)
Overall knowledge of incontinence surgery (*n* = 89)	No knowledge			55 (62%)
	Knowledge of at least one surgical therapy			34 (38%)
Knowledge of surgical options	Sling surgeries (*n* = 86)			21 (25%)
	Adjustable Systems (*n* = 86)			10 (12%)
	Artificial sphincter (*n* = 88)			22 (25%)
Do you speak with your urologist about your incontinence issues? (*n* = 94)	Yes, my urologist asks regularly			57 (60%)
	Yes, my urologist asks irregularly			12 (13%)
	Yes, but I have to bring it up myself			17 (18%)
	No, but I wish my urologist would bring it up with me			8 (9%)
	No, because I don’t want to talk about it			0 (0%)
Whom do you talk to about your incontinence issues? (*n* = 99)	Partner			68 (69%)
	Relatives/friends			31 (31%)
	Urologist			89 (90%)
	General practitioner			42 (42%)
	Other doctors			22 (22%)
	Local support group			6 (6%)
	Online forums			11 (11%)
Who informed you about continence therapy? Moreover, was this information important?	Treating urologist (*n* = 96)	No information		11 (12%)
		Unimportant information		7 (7%)
		Important information		78 (81%)
	General practitioner (*n* = 80)	No information		47 (58%)
		Unimportant information		11 (14%)
		Important information		22 (28%)
	Operating clinic (*n* = 86)	No information		40 (47%)
		Unimportant information		3 (3%)
		Important information		43 (50%)
	Support group (*n* = 80)	No information		69 (86%)
		Unimportant information		6 (8%)
		Important information		5 (6%)
	Partner (*n* = 88)	No information		36 (41%)
		Unimportant information		13 (15%)
		Important information		39 (44%)
	Relatives/friends (*n* = 81)	No information		51 (63%)
		Unimportant information		16 (20%)
		Important information		14 (17%)
	Internet (*n* = 80)	No information		51 (63%)
		Unimportant information		14 (18%)
		Important information		15 (19%)
	TV/Radio (*n* = 83)	No information		52 (63%)
		Unimportant information		18 (22%)
		Important information		12 (15%)

### Sources of information on continence management

Urologists were the most frequently cited source of information regarding incontinence (81%, 78/96), followed by treating hospital (50%, 43/86) and partner (44%, 39/88). Internet forums (19%, 15/80), relatives (17%, 14/81), television/radio (15%, 12/83), and self-help groups (6%, 5/80), were mentioned less frequently. 60% (57/99) of patients reported regular conversations about their SUI, whereas 18% (17/99) had to raise the topic themselves during medical consultations (Table 2).

### Knowledge of surgical treatment options and associated factors

Awareness of surgical treatment options for SUI was limited. Only 12% (10/86) were aware of adjustable systems such as Adjustable Continence Therapie (ProACT) or Adjustable Transobturator Male System (ATOMS), 24% (21/86) of the participants were aware of male sling procedures, and 25% (22/88) were aware of artificial urinary sphincters. Overall, 62% (55/89) reported no knowledge of any surgical option for managing incontinence (Table 2). Patients with prior knowledge of surgical treatments were younger (63.9 ± 8.1 vs. 68.6 ± 5.8; *p* = 0.002), used fewer pads (*p* = 0.04), and those participating in a local support group were more frequently informed about surgical therapies (5/6 vs. others; *p* = 0.02; Suppl. Table 2).

### Barriers to treatment uptake

**Fig. 1:**
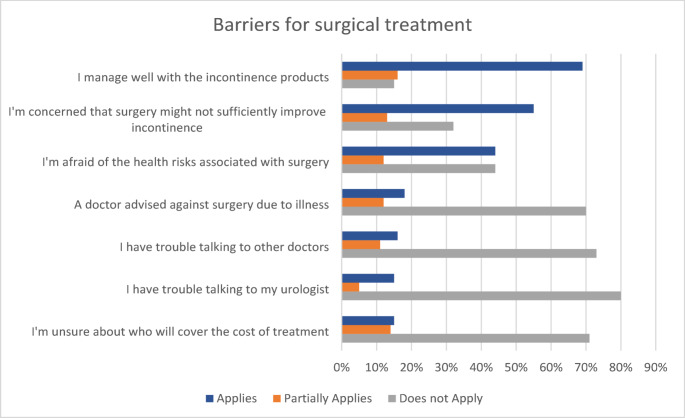
Barriers to seeking surgical treatment

The most frequently mentioned barriers to seeking surgical treatment for incontinence were satisfactory coping with absorbent products (69%, 62/90), doubts about the effectiveness of surgical procedures (55%, 49/90) and concerns regarding potential health risks associated with surgery (44%, 40/91) (Fig. 1 & Suppl. Table 1).

## Discussion

Our study reveals a profound and alarming knowledge deficit regarding the management of SUI. Awareness of basic incontinence aids is extremely low, while an overwhelming 62% (55/89) had no awareness of any surgical treatment options. Younger age, using fewer pads, and participation in support groups were associated with better awareness of treatment options for incontinence. The three most common reasons for not pursuing further treatment were adequate coping with continence aids (69%, 62/90), doubts about the effectiveness of surgery (55%, 49/90), and fear of health-related risks (44%, 40/91).

Although many patients report significant impairment due to post-prostatectomy incontinence (PPI), surgical intervention rates remain disproportionately low. Current estimates suggest that only 2.5% to 3.9% of patients undergo incontinence surgery after prostate cancer treatment [7, 12, 13], despite a considerably higher prevalence of persistent incontinence symptoms. This raises the question of what underlies the inadequate care. Similar patterns of low surgical intervention rates for PPI have been reported internationally, indicating that undertreatment is not unique to the German setting [13, 20, 21]. Our study reveals a substantial knowledge gap: 62% of respondents (55/89) reported no awareness of surgical treatment options for incontinence. This knowledge gap is associated with an older age and a higher pad use (Suppl. Table 2). Even more concerning is the limited awareness of continence aids, which can significantly improve quality of life without requiring surgical intervention [8, 22]. However, as previous studies have shown, the provision of aids and related counselling remains insufficient [23].

Low public awareness and persistent stigma surrounding male incontinence may discourage help-seeking behaviour [24]. This is especially problematic given that urologists routinely monitor patients as part of their prostate cancer aftercare. In our cohort, 90% of the patients discussed incontinence with their urologist, and 81% reported receiving important information. However, the consistency and quality of information appear to vary. Although communication frequency did not differ significantly between patients with and without knowledge of treatment options, this discrepancy suggests that regular contact with a specialist alone is not enough to ensure informed decision-making. Counselling was mainly provided by outpatient urologists. Structured education may include clinic-based counselling, written decision aids and digital formats; however, these formats were not explicitly assessed in this present cohort. In parallel to such educational approaches, primary care may also play an important role. Strengthening continence awareness in general practice and establishing clear referral pathways to urologists with functional expertise could improve early counselling and more timely access to appropriate treatment.

Decision-making quality improves significantly when patients feel well informed, knowledgeable, and supported in clarifying their values—conditions that are often not met in routine care settings [25]. Interestingly, different findings emerged regarding the treatment of ED in the same cohort, where communication with the urologist did have a measurable impact on care quality [17].

With regard to earlier studies, our findings confirm and extend existing evidence on the substantial gaps in patient knowledge and care related to urinary incontinence [26, 27]. Part of the problem may lie in the patients’ own underreporting of their condition [28]. Notably, we found no significant associations between knowledge or care gaps and sociodemographic factors such as education level, income, or insurance status.

To further explore the reasons behind the treatment gap, patients were asked about potential barriers to care. One of the most frequently cited reasons was satisfaction with current continence aids (69%, 62/90), indicating that many patients experience a good quality of life and do not feel a need for further intervention. This aligns with previous findings from the HAROW study, in which 59% of incontinent patients who had not undergone surgery reported little to no impact of incontinence on their quality of life [12]. For these individuals, surgical treatment may not be necessary. However, the survey on barriers also revealed knowledge gaps that could be addressed through proper medical counselling: 55% expressed doubts about the effectiveness of surgical procedures, 44% had concerns about potential health risks associated with surgery, and 15% were uncertain about the coverage of costs. These misconceptions represent avoidable barriers to informed decision-making. Although health-economic studies specifically on PPI are limited, existing analyses show that urinary incontinence is associated with substantial indirect social costs, including productivity loss and reduced workforce participation [29].

Medical reasons may be another factor in the decision not to pursue surgery, but they appear to affect only a minority of patients; in our study, only 18% reported medical reasons for non-treatment. This finding is consistent with the HAROW study, which revealed no significant associations between treatment decisions and age or Charlson Comorbidity Index scores, suggesting that older or sicker patients are not disproportionately excluded from care [12].

Interestingly, analysis of communication patterns revealed that, alongside urologists and partners, self-help groups played a significant role in patient discussions. Among patients who reported prior knowledge of surgical treatment options, 5/6 (83%) cited self-help groups as a source of information. This suggests that peer support structures may help compensate for deficits in formal counselling by providing accessible, experience-based knowledge and emotional reassurance. Previous studies have shown that participation in (online) self-help groups can improve patients’ health literacy, coping, and treatment decision-making [30, 31]. These observations highlight the potential value of integrating peer-based support more systematically into post-prostatectomy follow-up. Complementary to this, the national continence organisation in Germany provides structured patient information and public awareness materials [23], and improving continence-related training during urology residency could further strengthen the consistency and quality of counselling.

### Strengths and limitations

This study has several strengths and limitations. A key limitation is the pooling of two studies with relatively small cohorts and partly differing questionnaires. While questions on knowledge, information behaviour, and barriers to treatment were identical, continence-specific quality of life was assessed differently, allowing only indirect conclusions from EORTC QLQ-C30 items. Recruitment may have been affected by non-responder bias, as participants were likely to have a higher health awareness than non-responders. Furthermore, all data were self-reported, introducing the risk of recall and social desirability bias, particularly regarding prior counselling and reasons for not pursuing treatment. Continence severity relied solely on self-reported pad use. In addition, OAB symptoms were not systematically assessed, limiting differentiation between pure stress incontinence and mixed incontinence. The long follow-up in the HAROW cohort may also have shaped patients’ attitudes. Recall bias may be particularly relevant in the HAROW cohort, as patients were asked to recall counselling from many years earlier. Moreover, the older age of the HAROW collective compared with the Dresden cohort could have facilitated coping with incontinence, though it does not explain the lack of earlier treatment uptake.

Despite these limitations, the study has notable strengths. It is the first to systematically examine reasons why men do not receive incontinence therapy after RP, combining prospective data from the multicentre HAROW study with a contemporary cross-sectional cohort. The HAROW cohort encompassed patients treated at 114 different institutions, representing about one-fourth of all German RP providers and thereby reducing bias from individual practice patterns. Importantly, the study provides a detailed analysis of patients’ awareness of treatment options and their specific concerns regarding surgical interventions. These findings yield valuable insights into informational and emotional barriers and highlight critical opportunities for improving patient-centred care.

## Conclusion

This study reveals a substantial gap in knowledge regarding continence aids and surgical treatment options among men with SUI after RP. In addition to limited knowledge, fear-related concerns hinder treatment uptake. These findings demonstrate that the low rate of continence surgery is not solely due to clinical ineligibility but is strongly associated with preventable barriers in patient education and communication. Improving continence care will require not only treatment expertise but also a more open, proactive, and patient-centred dialogue—starting with the first consultation.

## Data Availability

No datasets were generated or analysed during the current study.
